# Reversible Zn^2+^ Insertion in Tungsten Ion-Activated Titanium Dioxide Nanocrystals for Electrochromic Windows

**DOI:** 10.1007/s40820-021-00719-y

**Published:** 2021-09-14

**Authors:** Yi Liang, Sheng Cao, Qilin Wei, Ruosheng Zeng, Jialong Zhao, Haizeng Li, William W. Yu, Bingsuo Zou

**Affiliations:** 1 MOE Key Laboratory of New Processing Technology for Non-Ferrous Metals and Materials, and Guangxi Key Laboratory of Processing for Non-Ferrous Metals and Featured Materials, School of Physical Science and Technology, Guangxi University, Nanning, 530004 People’s Republic of China; 2 Institute of Frontier and Interdisciplinary Science, Shandong University, Qingdao, 266237 People’s Republic of China; 3 Department of Chemistry and Physics, Louisiana State University, Shreveport, LA 71115 USA

**Keywords:** Electrochromism, Smart windows, TiO_2_, Doping, Zn^2+^-based electrochromic

## Abstract

Zinc-anode-based electrochromic devices (ZECDs) are emerging as the next-generation energy-efficient transparent electronics. We report anatase W-doped TiO_2_ nanocrystals (NCs) as a Zn^2+^ active electrochromic material. It demonstrates that the W doping in TiO_2_ highly reduces the Zn^2+^ intercalation energy, thus triggering the electrochromism. The prototype ZECDs based on W-doped TiO_2_ NCs deliver a high optical modulation (66% at 550 nm), fast spectral response times (9/2.7 s at 550 nm for coloration/bleaching), and good electrochemical stability (8.2% optical modulation loss after 1000 cycles).

**Supplementary Information:**

The online version contains supplementary material available at 10.1007/s40820-021-00719-y.

## Highlights

- A reversible Zn^2+^ insertion in anatase TiO_2_ nanocrystals is reported for the first time.
- This is the first report regarding TiO_2_ for zinc-anode-based electrochromic devices, which will subsequently broaden its applications to zinc-ion electrochemical cells.
- A prototype device based on the TiO_2_ nanocrystals delivers a high optical modulation, fast response times, and robust electrochemical stability.

**Supplementary Information:**

The online version contains supplementary material available at 10.1007/s40820-021-00719-y.

## Graphical abstract

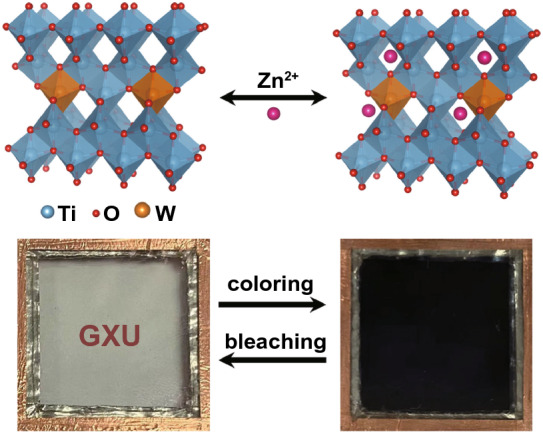

## Introduction

Electrochromic smart windows can alter the solar radiation transmittance by a small applied voltage according to the weather conditions and/or personal preferences, which reduces the energy consumption of lighting and cooling/heating of a room [1–9]. The basic working principle of electrochromism is that the color or optical properties of certain electroactive materials change reversibly by redox reactions through intercalation/delamination of electrolyte ions [1, 5, 10, 11]. Monovalent H^+^ and Li^+^ are the most used electrolyte ions in current electrochromic devices [12, 13]. Due to the limited reserves and high reactivity of Li metal and the strong corrosion tendency of acidic H^+^, the use of these electrolytes will inevitably increase the preparation cost and/or shorten the operation lifetime, thus limiting the real-world applications of electrochromic devices [5, 14–16]. Multivalent cations (e.g., Al^3+^, Mg^2+^, Zn^2+^) are more promising for electrochromism because they provide multiple charges compared to the monovalent Li^+^ and H^+^ [17–23]. This, as a result, will lead to a decrease in the amount of inserted cations and is expected to enhance the electrochromic performance, including rapid switching times and good cycling stability [24, 25]. In addition, some of the multivalent ions are compatible with aqueous electrolytes, which shows great advantages in operational safety and low production cost [17, 20, 24, 26]. Therefore, multivalent metal ions are gradually considered as superior alternatives to construct high-performance electrochromic devices.

Among various multivalent metal ions, Zn^2+^ is regarded as superior to others to trigger the electrochromism due to its simplified preparation process and nontoxicity [15, 20, 25, 27]. Furthermore, the relatively low redox potential of Zn^2+^/Zn (−0.763 V versus standard hydrogen electrode), which shows good compatibility with pH-neutral aqueous electrolytes and thus inhibiting the occurrence of hydrogen evolution reaction [28–32]. As a result, the pH value of the electrolyte will be stable during the device cycling. This, therefore, will significantly enhance the cycling stability of the devices [30, 33]. All these merits enable the ZECDs to become the state-of-the-art focus in the electrochromic community [15, 16, 20, 26, 27, 29, 34]. However, due to the high polarization and narrow voltage window characteristics of Zn^2+^ electrochemical reactions [29, 35], it is challenging to find a suitable electrochromic material as the host for the intercalation of Zn^2+^. To date, the existing ZECDs, almost all utilizing the classical electrochromic material (*i.e.*, WO_3_) as the cathode [16, 20, 27, 34], suffer from the corrosion of WO_3_ in aqueous acidic electrolyte. Therefore, it is of significant urgency to explore other cathodic electrochromic materials that can provide the same coloration ability while possessing a better electrochemical stability thanWO_3_ for a better implementation of the smart windows in practice.

Titanium dioxide (TiO_2_) is a cathode material with excellent physical, chemical stability, and acid resistance, which has been proved to have excellent electrochromic properties in Li^+^-based electrolytes [4, 12, 13, 36–39]. However, there is still no report regarding TiO_2_ for Zn-ion electrochemical cells from both the electrochromic and battery community yet. This is mainly attributed to the fact that divalent Zn^2+^ possesses a larger size and stronger coulombic ion lattice interactions than monovalent cations of Li^+^. As theoretically predicted from the thermodynamic mechanism, reducing the intercalation energy of ions can activate the reversible electrochemical behavior of known materials [40]. For example, the intercalation energy of Mg^2+^ and Al^3+^ is significantly reduced by introducing Ti vacancy into anatase TiO_2_, and thus reversible multivalent ion batteries with high efficiency and high capacity can be built [18]. Further exploration based on this work to activate Zn^2+^-triggered electrochemical (*e.g.*, electrochromic, energy storage properties) behavior of TiO_2_ should generate new superior performances in the electrochromic and battery applications.

In this work, we report the Zn^2+^-triggered electrochromic properties of TiO_2_ nanocrystals (NCs) for the first time. To implement the above-mentioned conjecture, colloidal W-doped TiO_2_ NCs were firstly prepared by a fluoride‐assisted controlled synthesis method. The as-synthesized doped TiO_2_ NCs showed a single anatase phase with a uniform size distribution. The electrochromic characterization in three-electrode measurements shows that the W-doped TiO_2_ NCs exhibit reversible electrochromic properties that are driven by Zn^2+^. The optical modulation range reaches as high as 77.6%, and the coloration and bleaching times are 10.4 and 2.2 s, respectively. Density functional theory (DFT) calculations confirm that that W doping TiO_2_ reduces the ion intercalation energy of Zn^2+^, thus activating the reversible Zn^2+^-triggered electrochromic properties of TiO_2_. The real-world applications of W-doped TiO_2_ NCs are also demonstrated in prototype electrochromic devices. The demonstrated devices confirm the good Zn^2+^ electrochromic performance with high optical modulation (66% at 550 nm), fast spectral response times (9/2.7 s at 550 nm for coloration/bleaching), and good electrochemical stability (the transmittance loss at 550 nm is 8.2% after 1000 cycles).

## Experimental Section

### Synthesis of Colloidal W-Doped TiO_2_ NCs

All preparations were performed in a Schlenk line system under flowing nitrogen (N_2_). Briefly, 1 mmol titanium ethoxide (technical grade), 0.1 mmol tungsten chloride, 8 mL 1-octadecene (90%,), 10 mmol 1-octadecanol (99%), 0.5 mL oleic acid (90%), 0.5 mL oleylamine (90%), and 0.4 mmol ammonium fluoride (NH_4_F, > 98%) were mixed in a 50 mL three-neck flask. After 20 min of vacuum degassing at 120 °C, the mixture was heated quickly to 280 °C in N_2_ atmosphere and kept at this temperature for 1 h for NC growth. After being cooled to ~ 60 °C, the solid NC samples were obtained by precipitation with acetone as well as a centrifugal procedure with 7000 rpm for 2 min. After that, the supernatant solution was discarded and the precipitation was re-dispersed in hexane for repeated centrifugal cycles (i.e., three times of the centrifugal procedure). Finally, the NCs were re-dispersed in toluene with a concentration of ~ 45 mg mL^−1^ for use. W-doped TiO_2_ with nominal W contents from 0 to 20 atom % was produced by varying the amount of tungsten chloride in the starting mixture while keeping all other synthesis parameters fixed.

### Fabrication of W-Doped TiO_2_ NC Film

A 2 × 2 cm^2^ FTO glass (10 Ω sq^−1^) was thoroughly cleaned with 2 vol% Hellmanex III solution for 5 min, rinsed with deionized water, and then washed with acetone and ethanol for 15 min each. 100 μL of the TiO_2_ NC solution (~ 45 mg mL^−1^) was then spin-coated onto the FTO glass at 1500 rpm for 30 s. After drying on a hot plate at 250 °C for 5 min, a second spin-coating was applied. The spin-coating processes were repeated for five times to build up the desired film thickness. The as-deposited doped TiO_2_ NC film on FTO glass was then heated in air at 400 °C for 50 min (5 °C min^−1^) to remove the organic ligands.

### Assembly of an Electrochromic Device

The demonstrated device was assembled with zinc foil as an anode sandwiched between two W-doped TiO_2_ (W4) NC film cathode electrodes. The three electrodes were assembled into an optical unit facing each other and separated by 3 M double-sided adhesive tape. A syringe was used to inject 1 M ZnSO_4_ electrolyte into the device. The assembled device was then sealed by transparent glue.

### Characterization

The composition, morphology, and structure of NCs were characterized by field emission scanning electron microscopy (FESEM, ZEISS SIGMA 500/VP) with energy-dispersive X-ray spectroscopy (EDS, using an INCA x-act attachment, Oxford), transmission electron microscope (TEM, FEI TECNAI G2 F30), X-ray powder diffraction (XRD, Rigaku corporation), Raman microscope (Raman, WITec, with a 532 nm Nd:YAG laser exciting source), and X-ray photoelectron spectroscopy (XPS, ESCALAB 250 XI +). The XPS peaks were curve-fitted by the XPSPeak41 software. Binding energies were corrected by referencing the C 1 s peak of adventitious carbon to 284.8 eV. In situ transmittance spectra of the films and devices were performed on an AVANTES spectrometer (AvaSpec-ULS2048CL-EVO). Electrochemical tests were carried out using an AUTOLAB PGSTAT204 electrochemical workstation.

### Electrochemical and Electrochromic Measurements

The electrochemical and electrochromic properties of doped TiO_2_ NC films were measured by using a custom-made three-electrode spectroelectrochemical cell connected to an AVANTES spectrometer. For the standard Zn^2+^ intercalation and de-intercalation measurements, a three-electrode in which Zn foil was used as counter electrode and reference electrode, and 1 M ZnSO_4_ was used as working electrolyte. The transmittance of an FTO glass that had undergone the same heat treatment as the working electrode in the same electrolyte was used as the baseline. All potentials were quoted with respect to a Zn^2+^/Zn standard. Switching time (τ) was defined as the time to achieve 90% of the full modulation in the specified potential range. Coloration efficiency (CE) was calculated by the formula: CE = ΔOD/ΔQ = log(T_b_/T_c_)/ΔQ, where ΔQ was the injected charge. All transmission spectra for different applied voltages were measured by the AVANTES spectrometer.

### Computation Details

The diffusion barriers of Zn^2+^ were calculated by climbing image nudged elastic band (NEB) method with a force-based optimization scheme. The structure optimization was implemented in the Vienna ab initio simulation package (VASP) based on DFT. To study single Zn^2+^ migration in anatase TiO_2_ and W-doped TiO_2_, supercell structures of 1 × 2 × 1 and 2 × 2 × 1 were constructed by using the unit cell of anatase TiO_2_. The atomic number of pure TiO_2_ is Ti_16_O_32_. In pure TiO_2_ with supercell structures of 1 × 2 × 1 and 2 × 2 × 1, one of Ti is replaced by W to construct W-doped TiO_2_ structure with the atomic number of Ti_7_WO_16_ and Ti_15_WO_32_. A Zn atom is adsorbed on the surface of Ti_16_O_32_ and diffused from one structural site to another. The same calculation method is used in Ti_7_WO_16_ and Ti_15_WO_32_. In detail, we give the calculated energy barrier of Zn^2+^ diffusion on the (001) surface, which takes the initial structure (0) as the zero point, the x-axis is the length of Zn atom in the Zn atom distance (0) structure, and the y-axis is the energy of each structure minus the energy of (0) structure, that is, if it is positive, the energy of this position is higher than (0), and if it is negative, it is lower than (0).

## Results and Discussion

### Preparation, Morphology, and Microstructure of W-doped TiO_2_ NCs

**Fig. 1:**
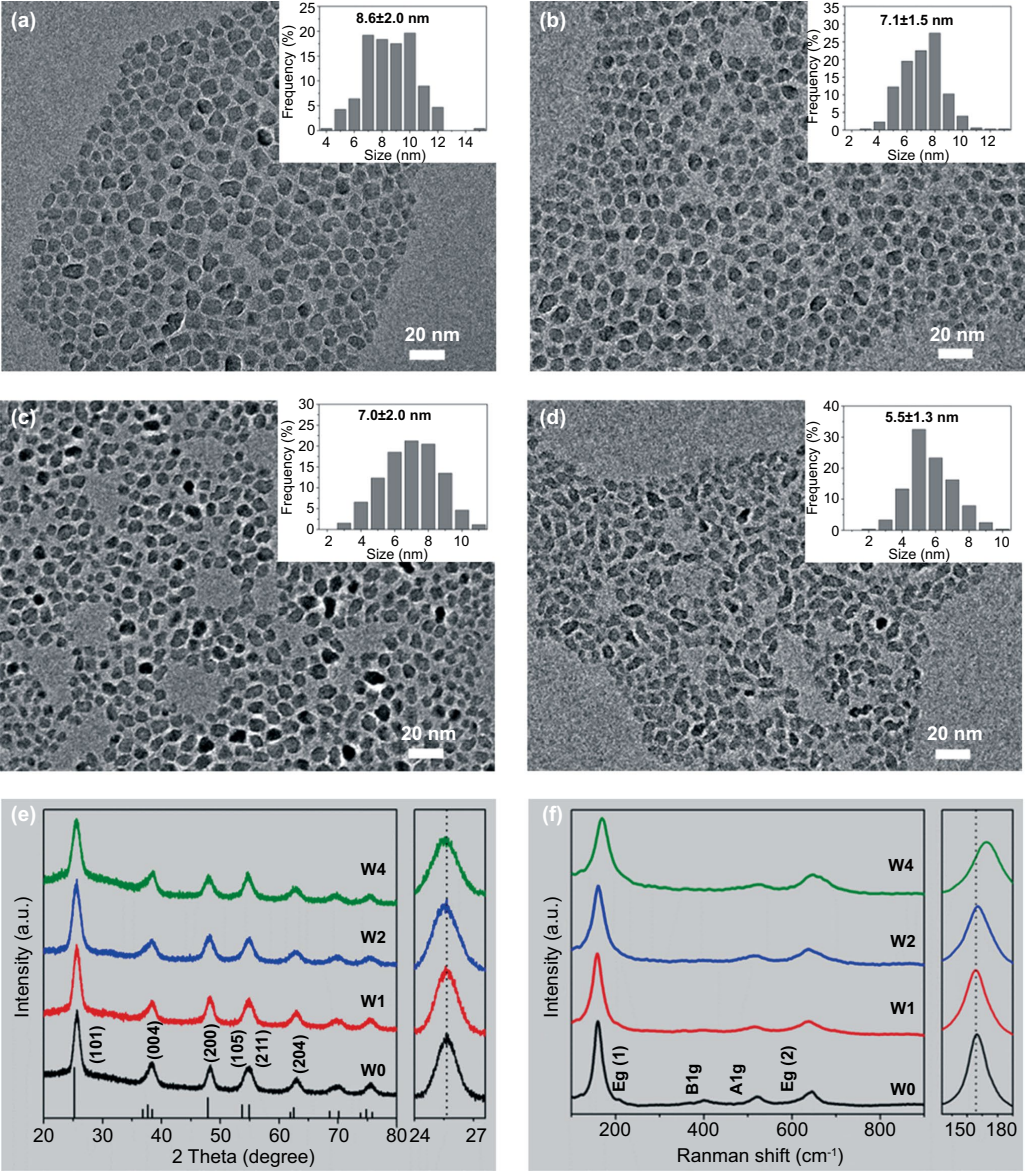
Morphologies and Structural evolution of W-doped TiO_2_ NCs with different W doping content. **a–d** are TEM images and size distributions (inserts) for W0, W1, W2, and W4, respectively. **e** XRD patterns. The right panel shows the magnified (101) diffraction peaks. **f** Raman spectra. The right panel shows the shift of E_g_ (1) peaks

A series of W-doped TiO_2_ colloidal NCs with different W contents were prepared through a modified fluoride‐assisted one‐pot method [37] by varying the W to Ti atomic ratio in the precursor mixture. Note that the oxygen anion in the TiO_2_ lattice prepared by this method is not replaced by the F anion (Fig. S1). The W to Ti precursor ratios of 0%, 5%, 10%, and 20% were denoted as W0, W1, W2, and W4. The energy-dispersive X-ray spectroscopy (EDS) results revealed that the actual W doping level of W0, W1, W2, and W4 were 0%, 1.09%, 2.38%, and 4.11%, respectively (Table S1). The content of W by EDS (a bulk analysis) is slightly lower than that by X-ray photoelectron spectroscopy (XPS, a surface analysis, Table S1), indicating that more W atoms are distributed on the surface of NC. The morphology of the as-synthesized W-doped TiO_2_ NCs is shown in Fig. 1a–d. It is found that all the samples were pseudospherical, with diameter distributions of 8.6 ± 2.0, 7.1 ± 1.5, 7.0 ± 2.0, and 5.5 ± 1.3 nm for W0, W1, W2, and W4, respectively. It is apparent that an increase in the dopant content decreased the NC size, which may be attributed to the effect of the W ions on crystal growth rate through surface charge modification [41].

Figure 1e compares the XRD patterns of W-doped TiO_2_ NCs with reference data (JCPDS No. 21–1272) of bulk TiO_2_ [37, 42]. All samples exhibit the anatase TiO_2_ structure with no signs of an impurity phase. It is found that the line width of diffraction peaks for all doped TiO_2_ NCs becomes wider with an increase in W concentration, suggesting that the size of NCs decreases with W doping and that is consistent with the TEM observation. The (101) diffraction peak in the right panel shift to lower 2θ values with an increase in W, which indicates that the unit cell expands with the incorporation of W ions into the TiO_2_ lattice [43]. This shift is also evidence of lattice doping where W ions with a larger effective ionic radius replacing smaller Ti ions [42]. The microstructure of W-doped TiO_2_ was further characterized by Raman spectroscopy. As shown in Fig. 1f, the typical anatase peaks at 159 cm^−1^ (E_g_), 522.6 cm^−1^ (A_1g_), and 646.2 cm^−1^ (E_g_) are observed in all samples [38, 44]. The absence of other Raman peaks in all doped samples further indicates that there is no other heterophase. The expanded view of the low-frequency E_g_ peak is shifted from 159.3 to 169.3 cm^−1^ with the increasing W doping level. Since the E_g_ peak is a characteristic Ti−O stretching mode, when Ti ions in the lattice are replaced by W ions, this shift can be used as another favorable evidence for W entering the lattice of TiO_2_ [44]. It has been reported that the ion radius of Ti^4+^ is 0.60 Å, while the ionic radii of the dopant ions are W^6+^(0.60 Å)/W^5+^(0.62 Å). The small difference between the dopant and host ionic radii is the reason why W replaces the Ti ions in the oxide lattice [42, 43]. These measurements confirm that the W ions have been successfully incorporated into the TiO_2_ lattice, and the produced W-doped TiO_2_ NCs present a single anatase phase.

### Zn^2+^-based Electrochemical and Electrochromic Performance of W-Doped TiO_2_ NCs

**Fig. 2:**
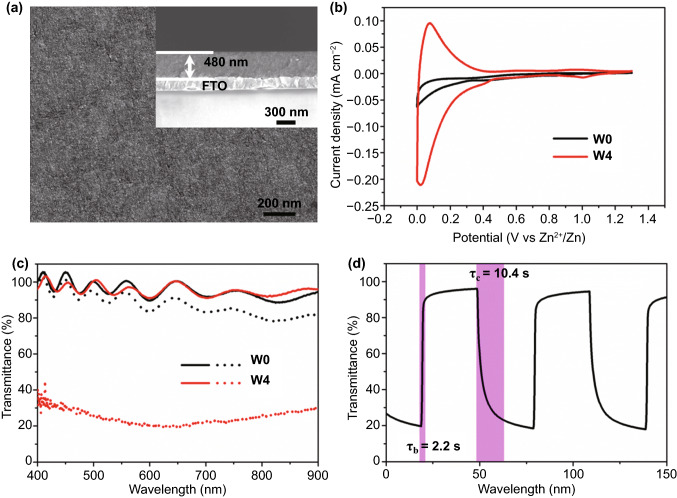
Characterizations of W-doped TiO_2_ NC films. **a** Surface and cross-sectional (inset) SEM images of a W4 film. **b** Voltammograms of the TiO_2_ and W4 NC films at 1 mV s^−1^ in the 0–1.3 V (vs. Zn^2+^/Zn) window in 1 M ZnSO_4_ aqueous electrolyte. **c** Optical transmittance spectra of the TiO_2_ and W4 NC films at fully colored (dot lines) and bleached (solid lines) states. **d** In situ optical transmittance of a W4 NC film at 550 nm in potential steps of 0–1.3 V

To evaluate the effect of W doping on the Zn^2+^ electrochromic properties of TiO_2_ NCs, these NCs were spin-coated on FTO glass and then heat-treated in the air up to 400 °C to remove surfactant and form transparent conductive NC film. Scanning electron microscopy (SEM, Fig. 2a) indicates that the W-doped TiO_2_ NC film is uniform and the thickness of the film is 480 nm. The Zn^2+^ electrochemical and electrochromic properties of doped TiO_2_ NC films were characterized by a three-electrode spectroelectrochemical cell, which is configured with 1 M ZnSO_4_ electrolyte and Zn sheets as counter electrode and reference electrode. To intuitively evaluate the effect of W doping on Zn^2+^ electrochromic, W0 and W4 were selected for comparative study in the main text, while the information of other samples was included in the Supporting Information (Figs. S2–S4). Figure 2b shows the cyclic voltammograms (CVs) of W0 and W4 films at a scanning rate of 1 mV s^−1^ in a potential range of 0–1.3 V (vs. Zn^2+^/Zn). It can be seen that the current density of W4 film is much higher than that of W0 film, indicating that W doping improves the Zn^2+^ electrochemical performance of TiO_2_ NCs (Fig. S2). Compared with TiO_2_ NCs, the Zn^2+^ diffusion coefficient in W-doped TiO_2_ NCs is significantly improved (Fig. S3), which further indicates that W doping in TiO_2_ NCs can activate the kinetics of Zn^2+^ electrochemistry. Figure 2c exhibits the transmittance spectra of W0 and W4 films under fully bleached (1.3 V for 30 s) and fully colored states (0 V for 30 s). The wavy transmittance spectra and the > 100% transmittance of the bleached electrochromic film are attributed to the constructive interference [3, 5]. For undoped TiO_2_ NC film, it is found that the optical transmittances between colored and bleached states change very little (*i.e.,* 5% at 550 nm). The poor electrochromic properties of pure anatase TiO_2_ NCs may be due to the strong Coulomb lattice interaction [24], which leads to the ineffective intercalation of Zn^2+^ ions into TiO_2_ lattice. While for the W-doped TiO_2_ NC film, although the transmittance of the bleached state is almost the same as that of undoped TiO_2_ NC film, the transmittance of the colored state is much lower than that of W0 film (Figs. S4 and S5). Since the different sizes of the W0 and W4 NCs, the electrochromic properties of pure TiO_2_ NCs with similar size to W4 were characterized to reveal the effect of NC size on the electrochromic properties. The two sizes of pure TiO_2_ NCs have the almost same spectral modulation range (Fig. S6), indicating that the size of NCs has a limited effect on the Zn^2+^ electrochromic properties. The above results therefore clearly indicate that W doping remarkably boosts the Zn^2+^ electrochromic properties of TiO_2_ NCs.

Figure 2d shows the dynamics in the electrochromic modulation of the optical response of the W-doped TiO_2_ NC film electrode. It is observed that the transmittance modulation of W-doped TiO_2_ film at 550 nm reaches 77.6%, and the coloration time (τ_c_) and bleaching time (τ_b_) are 10.4 and 2.2 s, respectively. The coloration efficiency of this film is 37.3 cm^2^ c^−1^ (Fig. S7), which is equivalent to the typical electrochromic properties of TiO_2_ [4, 8, 37]. The high transmittance modulation (77%) and response time (τ_b_/τ_c_ = 7.9/5.8 s) monitored at 633 nm were also observed (Fig. S8); all these properties can be compared with the best available Zn^2+^ electrochromic materials (Table S2) as well as the typical electrochromic properties of TiO_2_ (Table S3) reported. Since Li^+^-based electrolytes are still the most used electrolyte in an electrochromic device. Therefore, the comparison electrochromic properties of W-doped TiO_2_ NC films in Li^+^- and Zn^2+^-based electrolytes (the concentration of Li^+^ is 2 times higher than that of Zn^2+^) were performed (Fig. S9). The optical contrast in Li_2_SO_4_ electrolyte is 408% lower than that in ZnSO_4_ electrolyte, and the coloring efficiency of W4 obtained in Li_2_SO_4_ electrolyte is lower than that in ZnSO_4_ electrolyte, indicating that W-doped TiO_2_ NCs have better electrochromic properties driven by Zn^2+^ ions. These measurements demonstrate that the as-synthesized W-doped TiO_2_ NCs can be an alternative Zn^2+^ electrochromic material to meet the needs of next-generation smart windows.

### Mechanism Analysis of Zn^2+^-based Electrochromic of W-doped TiO_2_ NCs

**Fig. 3:**
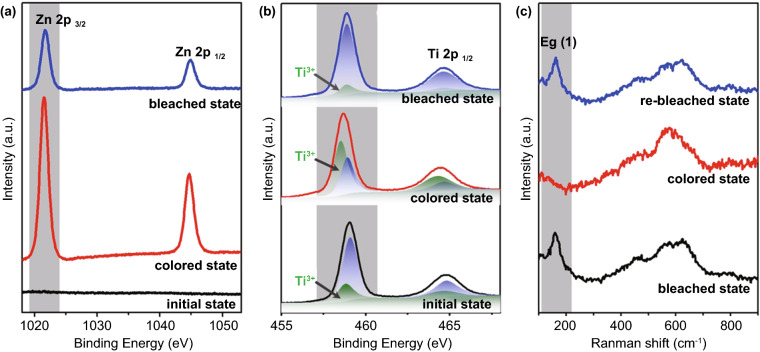
Structural evolution of a W-doped TiO_2_ NC film during the Zn^2+^ electrochromic process. **a–b** Ex situ high-resolution XPS spectrum of Zn 2p (**a**) and Ti 2p (**b**) in the initial, colored (0 V) and bleached (1.3 V) states. **c** In situ Raman spectra during the colored and bleached states

The ex situ XPS and in situ Raman spectra of W-doped TiO_2_ NC films were conducted to investigate the Zn^2+^ electrochromic mechanism. As shown in Fig. 3a, there is no Zn XPS signal in the initial electrode, but there is an obvious Zn 2p peak in the fully colored state, indicating that Zn^2+^ ions are successfully intercalated into the host of TiO_2_. The peak of Zn 2p still appears in the bleached state, which indicates that a portion of the intercalated Zn^2+^ ions is trapped in the “deep” sites with high-energy barriers during the electrochromic cycles. The trapped Zn^2+^ ions can be extracted from the “deep” Zn^2+^ sites by applying a high current under a stable voltage window of the electrolyte for real applications [12, 45]. The peak area of Zn 2p of the bleached state is far smaller than that of the coloration state, which confirms the (de-)insertion of Zn^2+^. Figure 3b shows that the Ti 2p_3/2_ peak at the initial state can be divided into two peaks, corresponding to Ti^3+^ 2p_3/2_ and Ti^4+^ 2p_3/2_, respectively, at 458.5 and 458.9 eV [8, 46]. The calculated area ratio of Ti^3+^ to Ti^4+^ is 1/3, which is consistent with the fact that the TiO_2_ matrix remains electrically neutral after Ti is replaced by high valence W. After being fully colored at 0 V, the peak of Ti 2p shifts to higher binding energy. The obvious change of binding energy can be attributed to the change of coordination environment of titanium with zinc and oxygen atoms. Due to the reduction in Ti^4+^ to Ti^3+^ in the process of Zn^2+^ ion insertion, the two peaks become wider, and the ratio of Ti^3+^ 2p increases. The area ratio of Ti^3+^ to Ti^4+^ is calculated to be 2/1. Therefore, the high optical transmittance contrast of the W-doped TiO_2_ NC films may be due to the reduction in Ti^4+^ to Ti^3+^ during the Zn^2+^ ions intercalation [47]. At the fully bleached state at 1.3 V, the Ti 2p spectra almost return to the initial state after the extraction of Zn^2+^. The coordination environment and valence state of W also changed during the electrochromic process (Fig. S10). However, the reduction ratio of W^6+^ is much lower than that of T^4+^, which indicates that W mainly contributes to the activation of the Zn^2+^-based electrochromic performance of the doped TiO_2_ system. Figure 3c shows the Raman spectra of the W4 electrode in different states. There are obvious anatase TiO_2_ E_g_ peak in the initial state and bleached state, but it disappears in the colored state, which indicates the coordination environment of Ti–O was changed in the Zn^2+^ intercalation process [44]. No new diffraction peak appears in the NC film at bleached and colored state (Fig. S11), suggesting that the anatase TiO_2_ phase is maintained throughout the electrochromic process. The above results show that in W-doped TiO_2_ NCs, due to the intercalation of Zn^2+^, the injected electrons are localized in Ti^4+^, resulting in the blue shift of absorption band, which leads to the obvious color change.

**Fig. 4:**
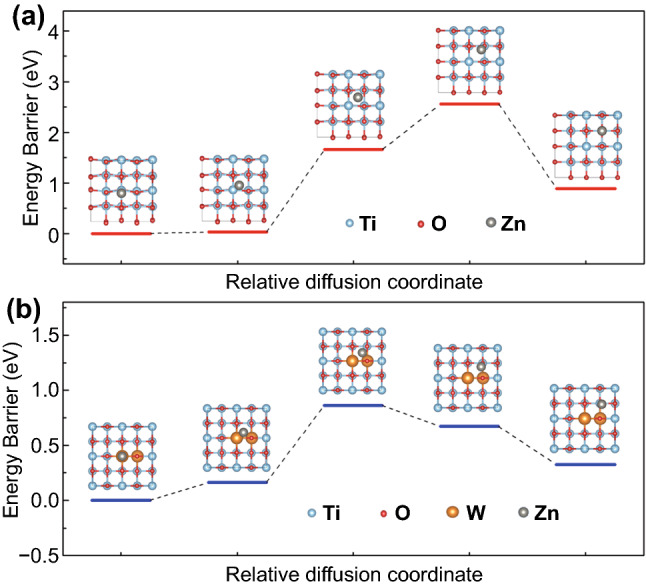
DFT calculations. The diffusion energy curves of isolated Zn^2+^ from one stable position to a neighboring one in the **a** TiO_2_ and **b** doped TiO_2_ with a W content of 4.2%. Insets show the possible Zn^2+^ insertion sites in TiO_2_ and W-doped TiO_2_ during the diffusion process

To reveal the influence of W doping on the Zn^2+^ electrochromic properties of activated TiO_2_, the diffusion barriers of Zn^2+^ in anatase TiO_2_ and W-doped TiO_2_ were further studied by DFT calculation [40, 48]. Figure 4 shows the diffusion energy curves of isolated Zn^2+^ from one stable position to a neighboring one in the TiO_2_ and W-doped TiO_2_. It can be seen that in anatase TiO_2_, the barrier energy required for the diffusion of a Zn^2+^ adsorbed on the surface from one structural site to another is 2.56 eV; while for W-doped TiO_2_ with a W content of 4.2%, the barrier energy required for the diffusion of adjacent structural sites is only 0.86 eV. The decrease in the Zn^2+^ diffusion barrier in W-doped TiO_2_ was also found at the doping level of 2.1% (Fig. S12), which may be due to the distortion of the TiO_6_ octahedron caused by the introduction of W dopant ions [18, 30, 49]. In general, this small diffusion barrier is more conducive to the diffusion of Zn^2+^ ions in the lattice, thus activating the Zn^2+^ electrochemical reaction of TiO_2_. The DFT calculation results are in good agreement with the above experimental results, indicating that W doping in anatase TiO_2_ is a facile way to improve the electrochemical kinetics of Zn^2+^ ions in the matrix and activate the Zn^2+^ electrochromic properties.

### Zn^2+^-based Electrochromic Performance of Demonstration Device

**Fig. 5:**
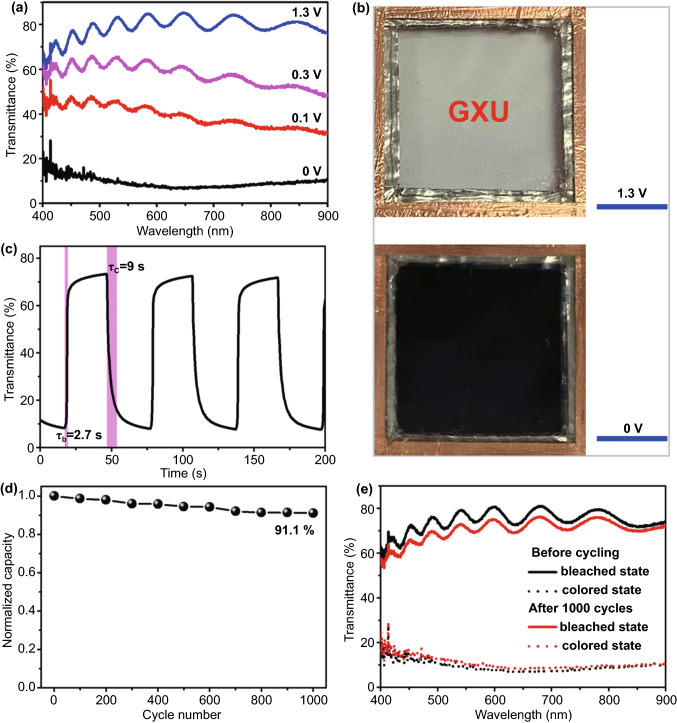
Electrochromic performance of demonstration device prepared by W4 sample. **a** Transmittance spectra at different operating voltages. **b** Photographs at fully bleached and colored state. The blue scare bar is 1 cm. **c** Real-time transmittance spectra of the device at 550 nm at 1.3–0 V. **d** Normalized charge capacity profile over 1000 voltammetric cycles at 20 mV s^−1^ between 0 and 1.3 V. **e** Optical transmittance spectra before and after 1000 cycles at fully bleached and colored states

For the demonstration of actual use of high performance of Zn^2+^ electrochromic, a prototype device is assembled by sandwiching zinc foil anode between two electrochromic W4 NC film electrodes and filled with 1 M ZnSO_4_ electrolyte [16]. Figure 5a shows that the demonstrative device delivers similar good Zn^2+^ electrochromic performance as in the three-electrode measurements, and the optical modulation range is as high as 66% at 550 nm. The optical modulation range of the device is slightly lower than that of the W4 single electrode measured in a three-electrode electrochemical cell due to the extra transmission loss of the second electrode in the sandwich structure. Figure 5b shows the corresponding photos of the device under fully bleached and colored states, showing a high coloration contrast of the bright and dark images. The optical response was characterized by in situ spectroscopy at 550 nm (Fig. 5c). The result discloses that the device shows fast spectral response and the coloration time τ_c_ and bleaching time τ_b_ are 9 and 2.7 s, respectively. The device also shows good bistability (Fig. S13). After the device being colored at 0 V for 30 s, the optical transmission is only increased by 5.7% and 5.1% at 550 and 633 nm after 3600 s, respectively. The cycle stability of the device was evaluated by CV at the commonly used scan rate of 20 mV s^−1^ [3, 50]. It is found that the cycle stability of the W-doped TiO_2_ NC thin film was good (Fig. S14), retaining 91.1% of its first-cycle (integrated) charge capacity after 1000 electrochemical cycles (Fig. 5d). Meanwhile, the high coloration contrast (Fig. S15) and the fast response time (Table S4) are also kept well in the electrochemical cycling, and the coloration contrast at 550 nm is 60.4% after 1000 cycles (Fig. 5e). This indicates that the decrease in transmittance modulation at 550 nm after 1000 cycles was just 8.2%. It is discovered that the surface morphology and structure of W4 film are almost unchanged after cycling, which shows a dense film structure comprised of anatase NCs (Figs. S16 and S17). This further indicates that TiO_2_ has excellent crystal structure stability and is suitable for the fabrication of ZECDs. These electrochromic performances indicate that the prototype device fabricated by W-doped TiO_2_ NCs is comparable to the state-of-the-art ZECDs (Table S5).

## Conclusions

In summary, we reported an effective method of W doping to boost Zn^2+^ electrochromic performance of anatase TiO_2_ NCs. The colloidal W-doped TiO_2_ NCs were prepared by a fluoride‐assisted synthesis. The as-synthesized W-doped TiO_2_ NCs were a single anatase phase with uniform size distribution. The electrochromic characterization in a three-electrode measurement showed that they exhibited reversible electrochromic properties driven by Zn^2+^. The optical modulation range at 550 nm reached 77.6%, and the coloration and bleaching times were 10.4 and 2.2 s, respectively. DFT calculations illustrated that W doping in TiO_2_ reduced the ion intercalation energy of Zn^2+^, thus activating the reversible Zn^2+^ insertion electrochromic properties of TiO_2_. The prototype ZECDs with W-doped TiO_2_ NCs were fabricated, which showed high optical modulation of 66% at 550 nm, fast spectral response time (9/2.7 s for coloration/bleaching), and good electrochemical stability (the optical modulation at 550 nm was only 8.2% after 1000 cycles). This work demonstrates that the W-doped TiO_2_ NCs are a promising Zn^2+^-active electrochromic material and also suggests that TiO_2_ can be a promising material for other electrochemical applications such as zinc ion batteries.

## Supplementary Information

Below is the link to the electronic supplementary material.

Supplementary file1 (PDF 1569 kb)
