# Correction to: Reversible Zn^2+^ Insertion in Tungsten Ion Activated Titanium Dioxide Nanocrystals for Electrochromic Windows

**DOI:** 10.1007/s40820-021-00765-6

**Published:** 2021-11-26

**Authors:** Yi Liang, Sheng Cao, Qilin Wei, Ruosheng Zeng, Jialong Zhao, Haizeng Li, William W. Yu, Bingsuo Zou

**Affiliations:** 1 MOE Key Laboratory of New Processing Technology for Non-Ferrous Metals and Materials, and Guangxi Key Laboratory of Processing for Non-Ferrous Metals and Featured Materials, School of Physical Science and Technology, Guangxi University, Nanning, 530004 People’s Republic of China; 2 Institute of Frontier and Interdisciplinary Science, Shandong University, Qingdao, 266237 People’s Republic of China; 3 Department of Chemistry and Physics, Louisiana State University, Shreveport, LA 71115 USA

## Correction to: Nano-Micro Lett. (2021) 13:196 10.1007/s40820-021-00719-y

In the original version of this article, there is an unintentional wrong description of the x-coordinate in Fig. 2d. The correct Fig. 2 is provided in this correction. This changes none of the conclusions of the manuscript.

**Fig. 2:**
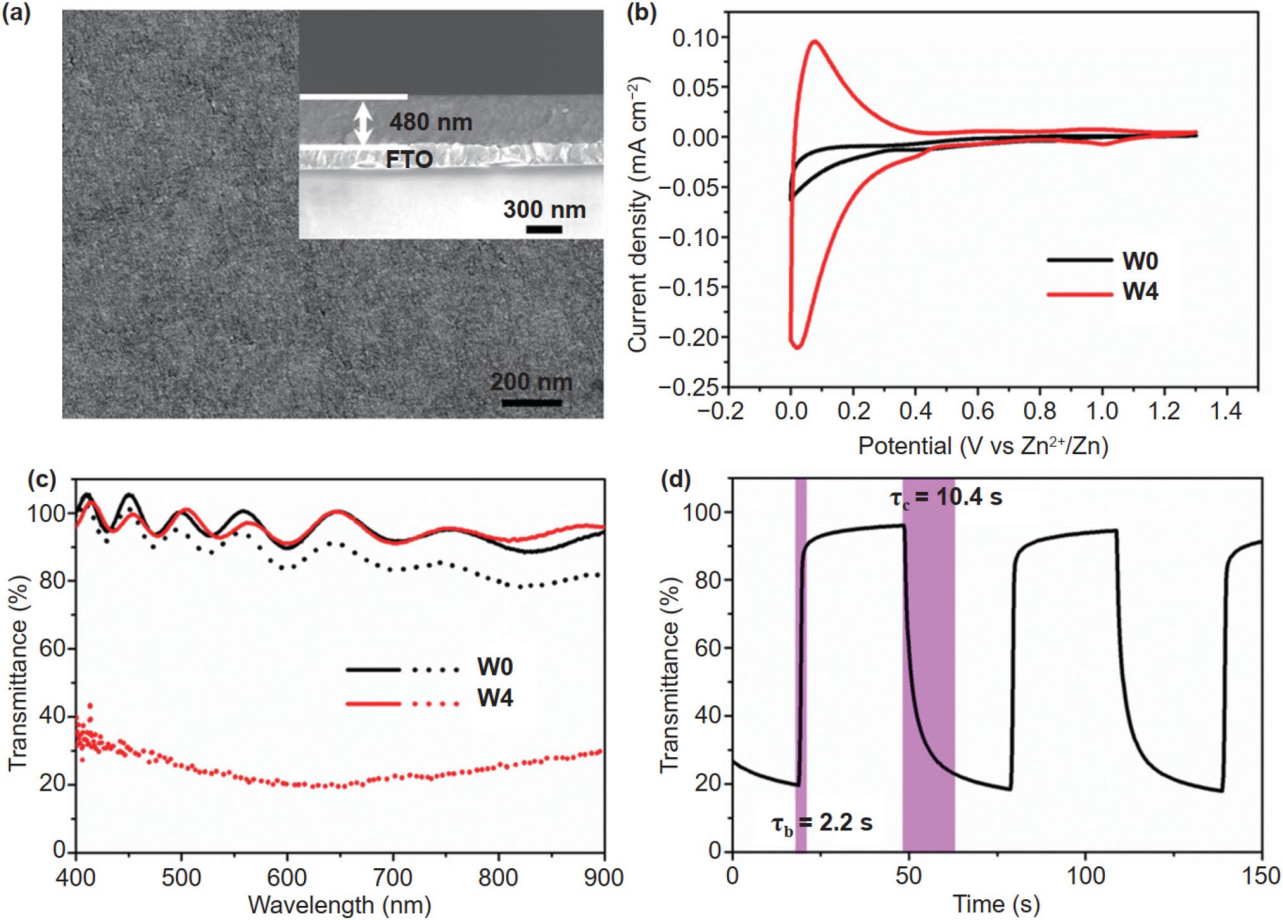
Characterizations of W-doped TiO_2_ NC films. **a** Surface and cross-sectional (inset) SEM images of a W4 film. **b** Voltammograms of the TiO_2_ and W4 NC films at 1 mV s^−1^ in the 0–1.3 V (vs. Zn^2+^/Zn) window in 1 M ZnSO_4_ aqueous electrolyte. **c** Optical transmittance spectra of the TiO_2_ and W4 NC films at fully colored (dot lines) and bleached (solid lines) states. **d** In situ optical transmittance of a W4 NC film at 550 nm in potential steps of 0–1.3 V

