# Supplementary material for: Reversible Zn2+ Insertion in Tungsten Ion-Activated Titanium Dioxide Nanocrystals for Electrochromic Windows
Source: Nanomicro Lett. 2021 Sep 14;13:196. doi: 10.1007/s40820-021-00719-y (PMC8440694; doi:10.1007/s40820-021-00719-y)
Supplement: Supplementary file 1 — Supplementary file1 (PDF 1569 kb) [file 40820_2021_719_MOESM1_ESM.pdf]

Supporting Information for

Reversible Zn^{2+} Insertion in Tungsten Ion Activated Titanium Dioxide Nanocrystals for Electrochromic Windows

Yi Liang[1], Sheng Cao[1, *], Qilin Wei[1], Ruosheng Zeng[1], Jialong Zhao[1], Haizeng Li[2, *], William W. Yu[3], and Bingsuo Zou[1, *]

[1]MOE Key Laboratory of New Processing Technology for Non-ferrous Metals and Materials, and Guangxi Key Laboratory of Processing for Non-ferrous Metals and Featured Materials, School of Physical Science and Technology, Guangxi University, Nanning, 530004, P. R. China

[2]Institute of Frontier & Interdisciplinary Science, Shandong University, Qingdao, 266237, P. R. China

[3]Department of Chemistry and Physics, Louisiana State University, Shreveport, LA 71115, US

*Corresponding authors. E-mail: caosheng@gxu.edu.cn (S. C.); haizeng@sdu.edu.cn (H. L.); zoubs@gxu.edu.cn (B. Z)

Supplementary Tables and Figures

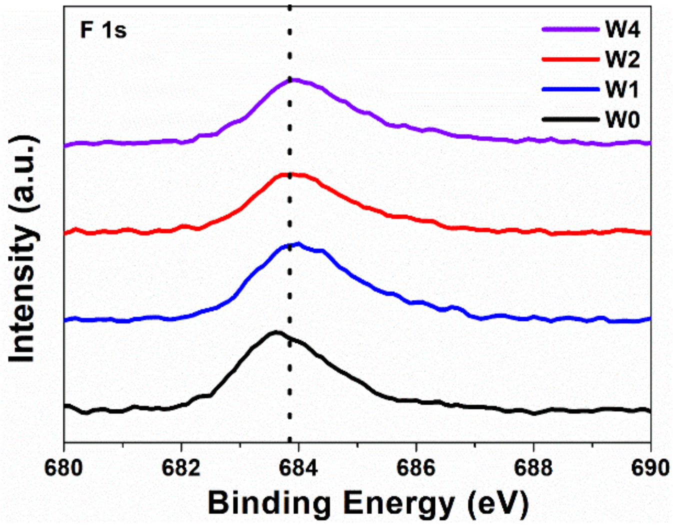

Fig. S1 F 1s peaks of W-doped TiO2 NCs with different doping level

As shown in Fig. S1, the F 1s XPS peaks of the doped TiO_2 NCs are all centered around 683.8 eV, which are attributed to the F-Ti bonds on the surface of the W-doped TiO_2 NC surface [S1, S2]. As there is no peak among the binding energy region between 688 and 689 eV, which indicates that the oxygen anion is not replaced by F anion in the lattice of TiO_2 [S3, S4].

Table S1 Chemical compositions of W-doped TiO_2 NCs with different W contents tested by EDS and XPS

Sample	W atom %		Ti atom %		O atom %	
	EDS	XPS	EDS	XPS	EDS	XPS
W0	0	0	33.7	35.56	66.3	64.44
W1	1.09	3.74	29.46	32.60	69.45	63.66
W2	2.38	4.70	26.71	30.50	70.91	64.80
W4	4.11	6.13	25.54	27.33	70.35	66.54

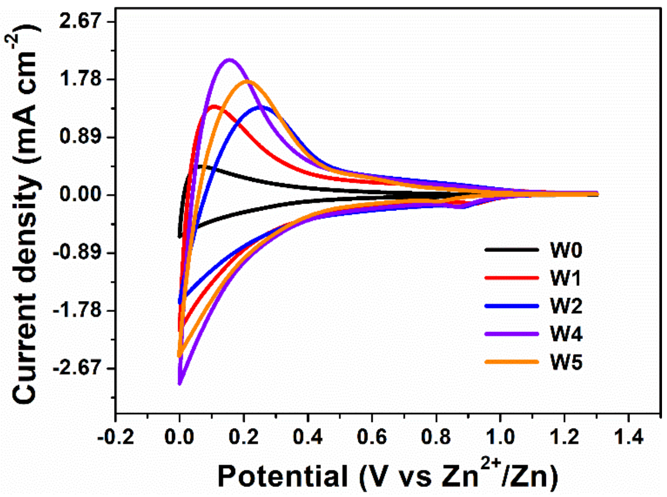

Fig. S2 Voltammograms of doped TiO_2 NC films with W nominal doing level of 0% (W0), 5% (W1), 10% (W2), 20% (W4) and 25% (W5). The scan rate is 20 mV s^{-1} in the 0-1.3 V (vs. Zn^{2+}/Zn) window in 1 M $ZnSO_4$ aqueous electrolyte.

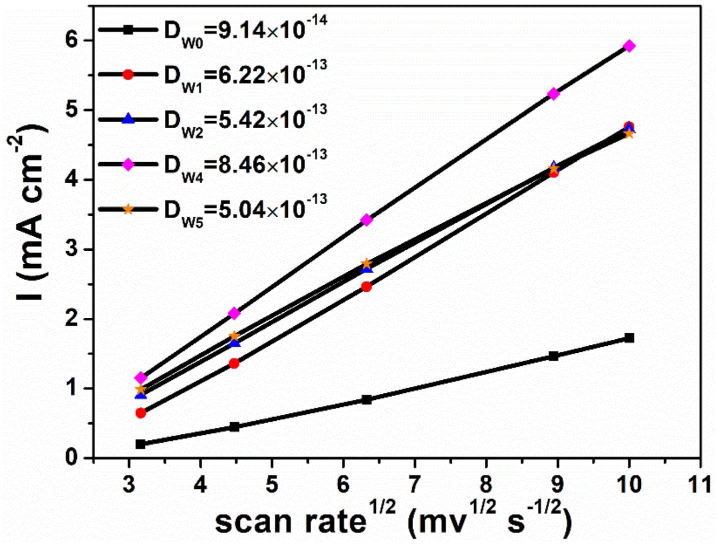

Fig. S3 Plot of I_P versus $v^{1/2}$ for the calculation of the effective diffusion coefficient Zn^{2+} in the electrochromic host

Figure S3 shows the relationship between peak current and scan rate of doped TiO_2 NC film with different W contents. The diffusion coefficients of Zn^{2+} in films thus can be calculated by Randles-Sevcik's equation [S5]. Accordingly, the diffusion coefficients of Zn^{2+} in W0, W1, W2, W4, and W5 are 9.14×10^{-14}, 6.22×10^{-14}, 5.42×10^{-14}, 8.46×10^{-14}, and 5.04×10^{-14} cm^2 s^{-1}, respectively. Compared with undoped TiO_2 NCs, the diffusion coefficient of Zn^{2+} in W doped TiO_2 NCs is higher, which indicates that W doping in TiO_2 NCs can activate the kinetics of Zn^{2+} based electrochromic.

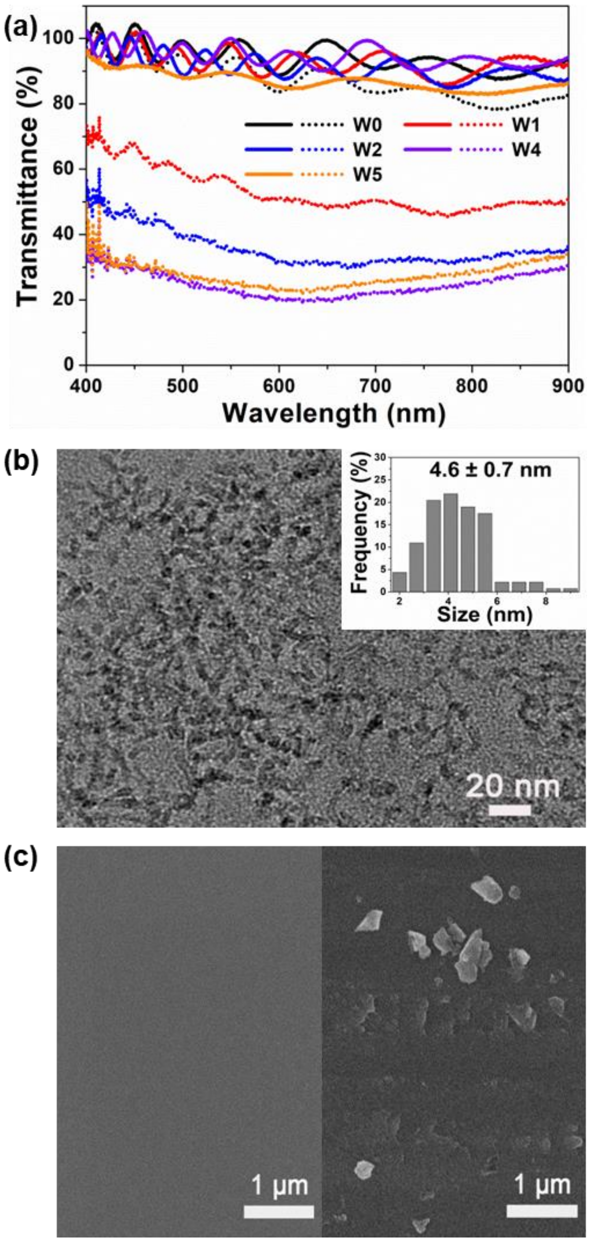

Fig. S4 (**a**) Optical transmittance spectra of doped TiO_2 NC films with W nominal doing level of 0% (W0), 5% (W1), 10% (W2), 20% (W4), and 25% (W5) at fully colored (dot lines) and bleached (solid lines) states. (**b**) TEM images of the W5 NCs. (**c**) SEM images of W4 (left) and W5 (right) NC film

Figure S4a shows that the transmittance modulation rates of W0, W1, W2, W4, and W5 films at 550 nm are 5%, 41.6%, 54.6%, 77.6%, and 64.6%, respectively. These results indicate that a moderate W doping level can significantly improve the Zn^{2+} active electrochromic properties of TiO_2 NCs, and the W4 film has the largest electrochromic modulation range due to its high Zn^{2+} electrochemical activity. The optical properties of W5 are worse than that of W4, which may be attributed to the decrease of grain width and increase of grain length in W5 NCs (Fig. S4b). Such a high aspect ratio makes the W5 NCs easier to be secondarily crystallized during the post-heat-treatment, and thus resulting in large particle size and non-uniformity size distribution in the W5 film (Fig. S4c) [S6-S8]. The presence of large particles may lead to the light scattering phenomenon, and thus resulting in a lower bleached transmittance of W5 film.

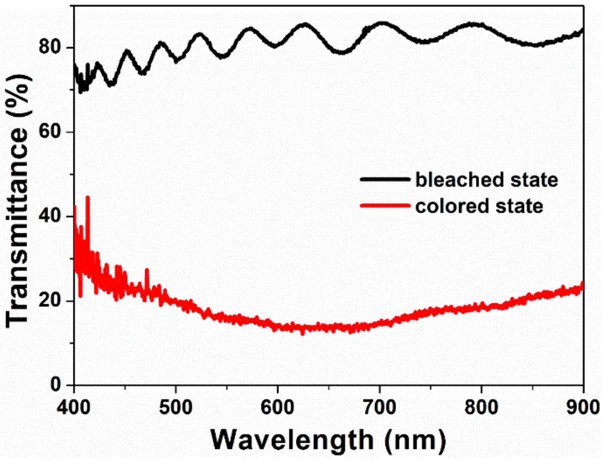

Fig. S5 Optical transmittance spectra of W4 film at bleached state and colored state where the electrolyte was selected as a baseline

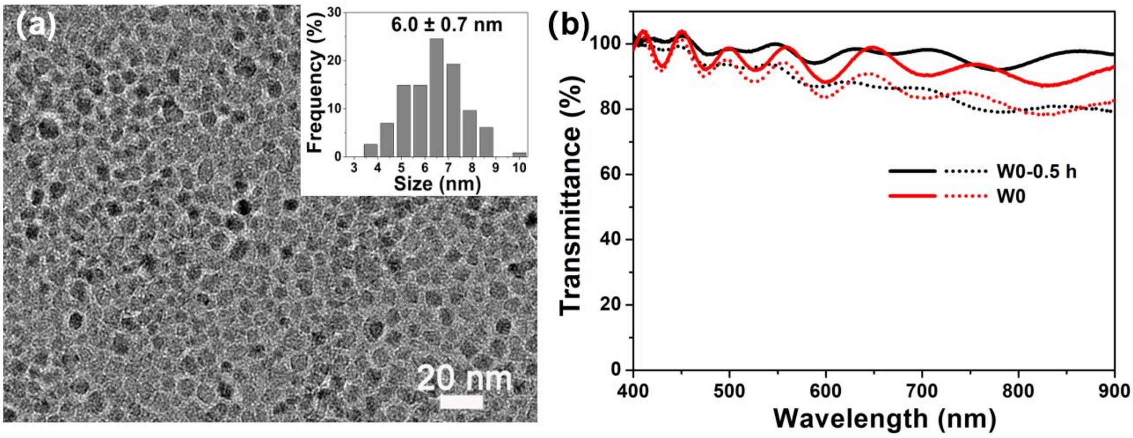

Fig. S6 (**a**) TEM image and size distribution (insert) of W0-0.5h NCs. (**b**) Optical transmittance spectra of W0 and W0-0.5h NC films at fully colored (dot lines) and bleached (solid lines) states in 1M $ZnSO_4$ electrolyte

To reveal the effect of size on the electrochromic properties of NC films, we shorten the growth time of TiO_2 NCs to 30 minutes, and the as-obtained samples are expressed as W0-0.5h. The morphology of W0-0.5h NCs is shown in Fig. S6a. It is found that the

sample is pseudospherical, with the diameter distribution of 6 ± 0.7 nm. The electrochromic properties of W0-0.5h NC films were measured and as shown in Fig. S6b. The transmittance modulation range at 550 nm is 6%, which is similar to that of W0 NC film with 5%. This indicates that the size of NCs has limited effect on the Zn^{2+} electrochromic properties in our case.

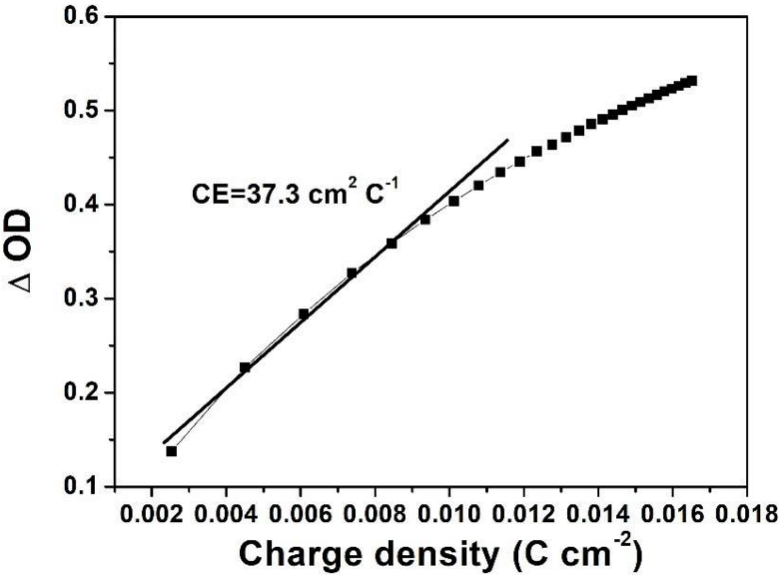

Fig. S7 Coloration efficiency of a W4 W-doped TiO_2 NCs at 550 nm

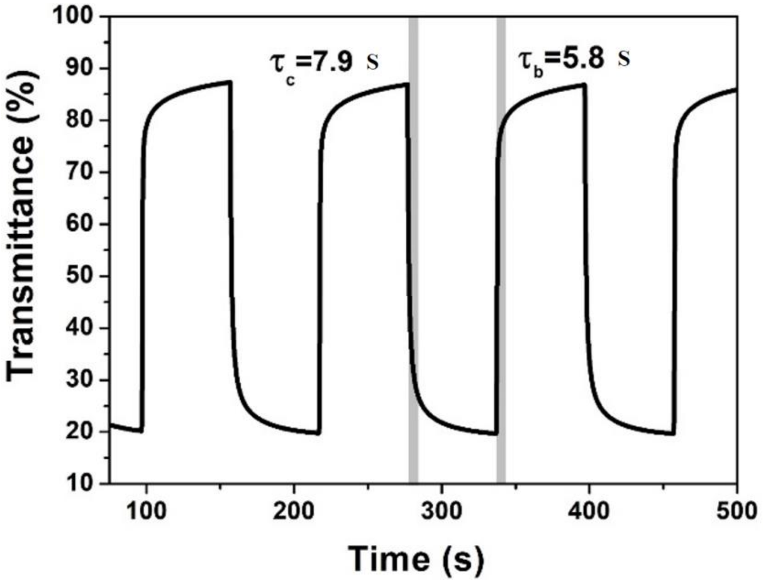

Fig. S8 Real-time transmittance changes of a W4 W-doped TiO_2 NC film at 633 nm in the 0-1.3 V window

Table S2 Performance comparison of Zn^{2+}-based electrochromic films

material	electrolyte	response time (s)	optical modulation (%)	Refs.
Ti-substituted tungsten molybdenum oxide nanowire bundles	1 M $ZnSO_4$	τ_c:14 (at 632.8 nm)	76 (at 632.8 nm) 69 (at 550 nm)	[S9]
WO_3 nanoparticles	1 M $ZnSO_4$	τ_b/τ_c:6.6/6.9 (at 632.8 nm)	82 (at 632.8 nm) 73 (at 550 nm)	[S10]
Sodium vanadium oxide nanorods	1 M $ZnSO_4$	τ_b/τ_c:25/12.6 (at 632.8 nm)	—	[S11]
WO_3 nanorods	1 M $ZnSO_4$	τ_b/τ_c:3/2 (at 550 nm)	81 (633 nm) 72 (550 nm)	[S5]
W-doped TiO_2 NCs	1 M $ZnSO_4$	τ_b/τ_c:2.2/10.4 (at 550 nm) τ_b/τ_c:5.8/7.9 (at 633 nm)	74.6 (at 550 nm) 77 (at 633 nm)	This work

Table S3 Typical electrochromic properties of TiO_2 reported in literatures

material	electrolyte	optical modulation (%)	response time (s)	cycle stability	CE (cm^2 C^{-1})	Ref.
Nb-doped TiO_2 NCs	1 M $LiClO_4$	72 (at 550 nm)	τ_b/τ_c: 105/10 (at 550 nm)	200 cycles	—	[S12]
Ta-doped TiO_2 NCs	0.5 M Li-TFSI	86.3 (at 550 nm)	τ_b/τ_c: 6.9/66.8 (at 550 nm)	1.3% decayed after 2000 cycles	33.2	[S1]
TiO_{2-x} NCs	1 M $LiClO_4$	95.4 (at 633 nm)	τ_b/τ_c: 9.6/35.1 (at 633 nm)	1.1% decayed after 2000 cycles	38.2	[S2]
Ta-doped TiO_2 NCs	1 M $LiClO_4$	89.1 (at 550 nm)	τ_b/τ_c:9.5/52.6 (at 550 nm)	0.2% decayed after 2000 cycles	29.7	[S13]

WO_3/TiO_2 nanotubes	0.1 M $HClO_4$	82 (at 600 nm)	τ_b/τ_c: ~1/~1 (at 600 nm)	—	—	[S14]
WO_3 nanoplates/ TiO_2 NCs	1 M $LiClO_4$	78* (at 600 nm)	τ_b/τ_c:6/6 (at 600 nm)	—	128.3	[S15]
W-doped TiO_2 NCs	1 M $ZnSO_4$	66 (at 550 nm)	τ_b/τ_c:2.7/9 (at 550 nm)	8.7% decayed after 1000 cycles	37.3	This work

* This data is the test value of reflectivity.

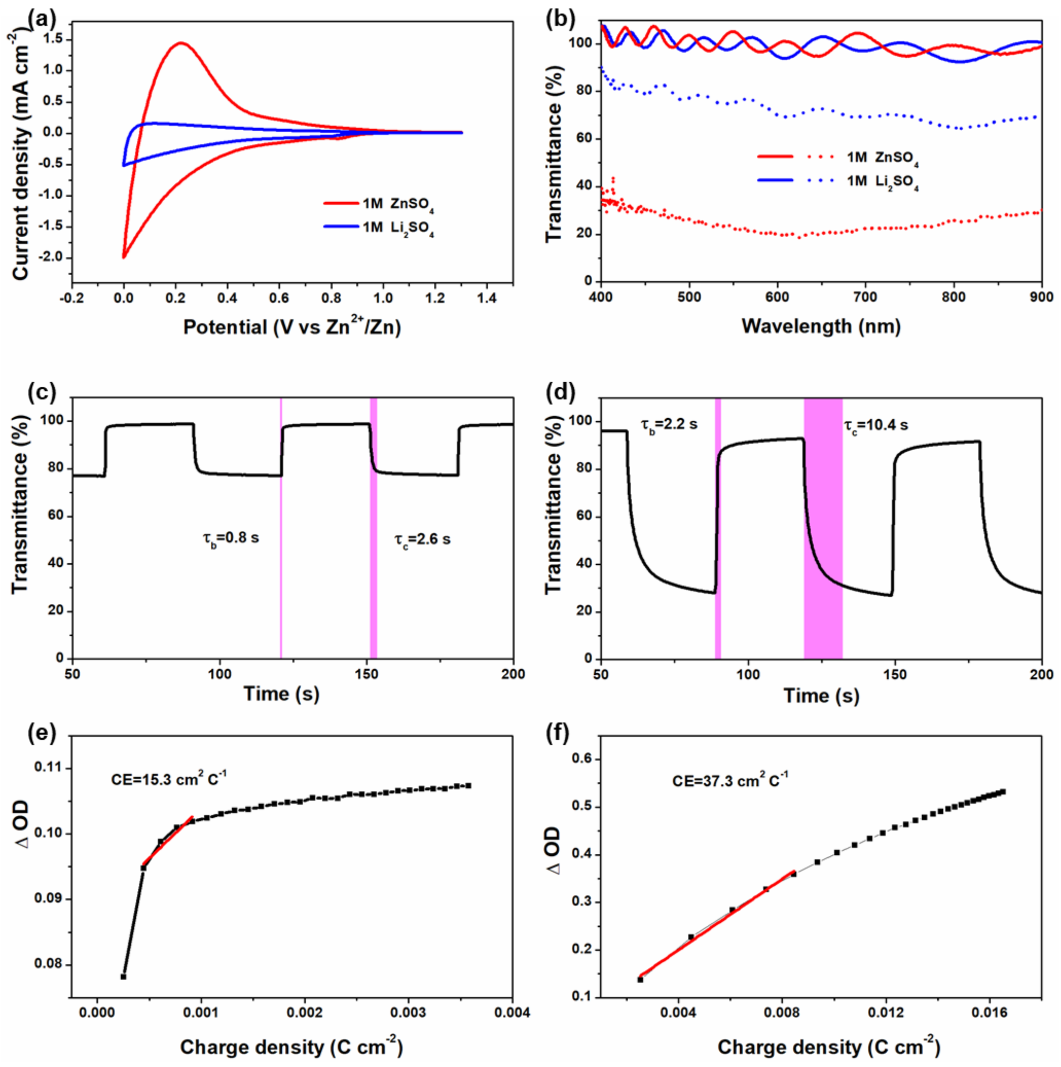

Fig. S9 Electrochromic properties of W4 films in 1 M Li_2SO_4 and $ZnSO_4$ electrolyte. (**a**) Voltammetric cycles at 20 mV s^{-1} in the 0-1.3V window. (**b**) Optical transmittance spectra of W4 NC films at fully colored (dot lines) and bleached (solid lines) states. (**c**) In-situ optical transmittance of W4 NC film at 550 nm in potential steps of the 0-1.3V window in Li_2SO_4 electrolyte. (**d**) In-situ optical transmittance of W4 NC film at 550 nm in potential steps of the 0-1.3V window in $ZnSO_4$ electrolyte. (**e**) Coloration

efficiency of a W4 film at 550 nm in Li_2SO_4 electrolyte. (**f**) Coloration efficiency of a W4 film at 550 nm in $ZnSO_4$ electrolyte.

Compared to the electrochromic performance achieved via using the $ZnSO_4$ electrolyte, the W4 electrode shows inferior electrochromic performance in the Li_2SO_4 electrolyte (Fig. S9a, b). As shown in Fig. S9a, the optical contrast tested in the Li_2SO_4 electrolyte is 408% lower than that tested in the $ZnSO_4$ electrolyte. Figure S9c-f that the response times and coloration efficiency of the W4 film in the electrolyte of Li_2SO_4 and $ZnSO_4$. The switching times of W4 film attained in Li_2SO_4 electrolyte are better than that tested in $ZnSO_4$ electrolyte, which is attributed to the fact that Li-ions are not effectively embedded into the lattice of TiO_2 in the same voltage window of 0-1.3V. In other words, the larger optical contrast will take longer switching times at the same switching speed and the W4 films have a larger optical contrast in a $ZnSO_4$ electrolyte. Obviously, the coloration efficiency of the W4 attained in Li_2SO_4 electrolyte is lower than that tested in $ZnSO_4$ electrolyte, which further confirms that Zn^{2+} is more electrochemically active to W4 films than Li^{+}. The above results further confirm the significance of developing Zn^{2+}-active electrochromic materials.

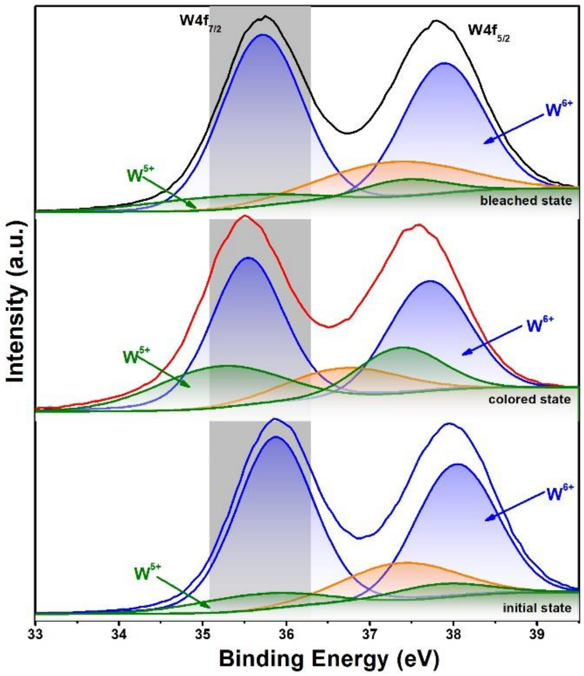

Fig. S10 Ex-situ high resolution XPS spectrum of W4f in the W-doped TiO_2 NC film at the initial, colored (0 V) and bleached (1.3 V) states

It is reported that the peak Ti 3p overlaps with the peak W4f, the Ti 3p peak was thus separated when we fitted the XPS spectrum of W [S16]. Figure S10 shows that the $W4f_{7/2}$ peak at initial state can be divided into two peaks, corresponding to W^{6+} $4f_{7/2}$ and W^{5+} $4f_{5/2}$ at 35.9 and 35.6 eV, respectively [S17]. The calculated area ratio of W^{5+} to W^{6+} is 1/7, which is consistent with the fact that the TiO_2 matrix remains electrically neutral after W replaced Ti [S18]. The XPS peak of W shifts to a low angle in the colored state, which may be due to the change of the coordination environment of W

with Zn and O atoms due to the insertion of Zn^{2+} ions [S10]. Due to the reduction of W^{6+} to W^{5+} in the process of Zn^{2+} ions intercalation, the area ratio of W^{5+} to W^{6+} is increased to 3/7. At the fully bleached state at 1.3 V, the W4f spectra almost return to the initial state after the deintercalation of Zn^{2+}. The reduction ratio of W^{6+} is much lower than that of T^{4+}, which indicates that W mainly plays the activate Zn^{2+} based electrochromic role in the doped TiO_2 system.

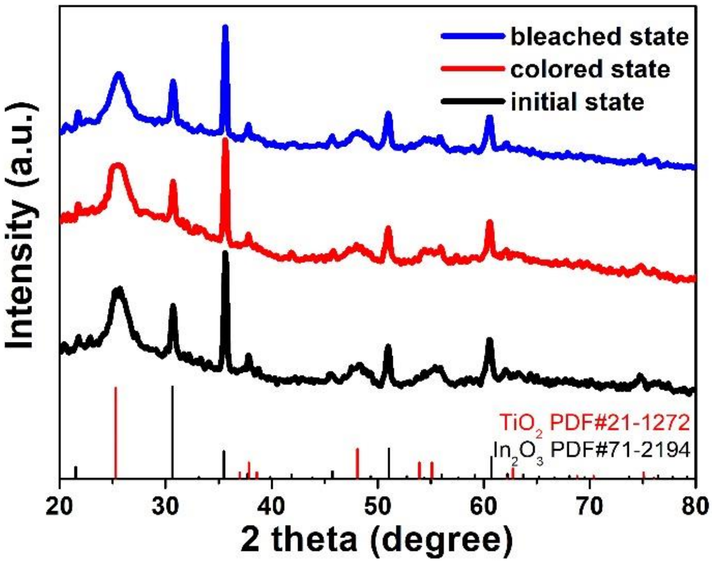

Fig. S11 Ex-situ XRD of W4 film during the Zn^{2+} active electrochromic process in the initial, colored (0 V) and bleached (1.3 V) states. In order to obtain strong XRD diffraction signal of NC film, ITO glass with weak XRD diffraction intensity was selected as the substrate for testing.

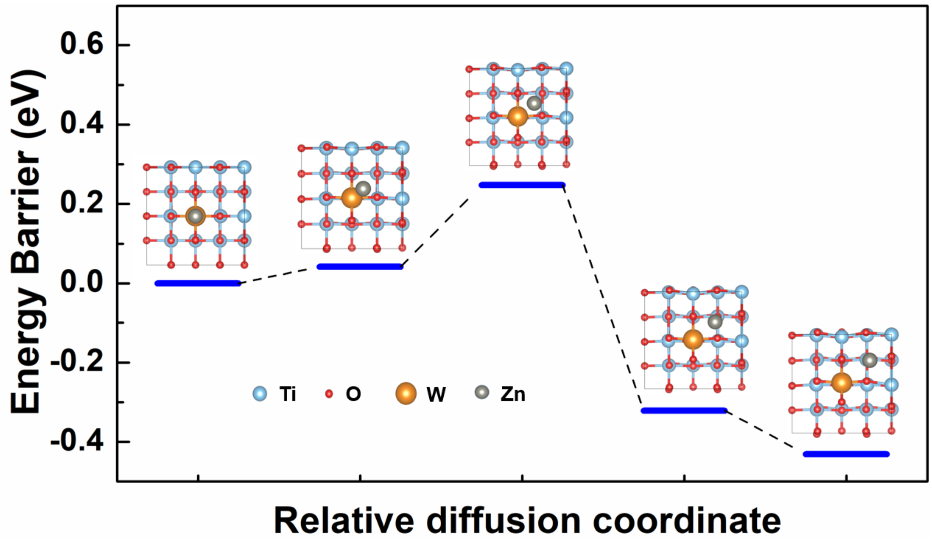

Fig. S12 The diffusion energy curves of isolated Zn^{2+} from one stable position to a neighboring one in the doped TiO_2 with W content of 2.1%

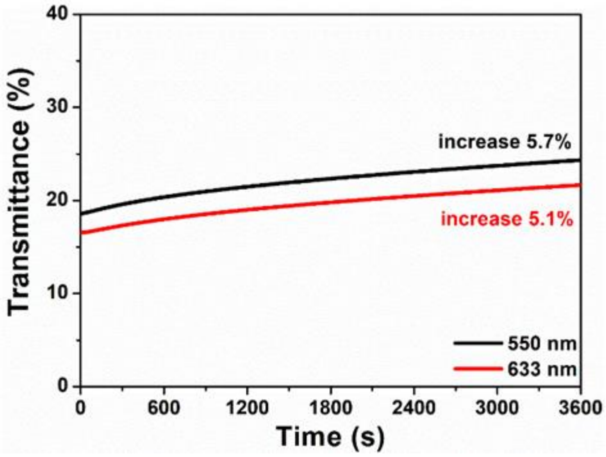

Fig. S13 Transmittance changes at 550 and 633 nm under the open-circuit condition after the W4 device was biased at 0 V for 30 s

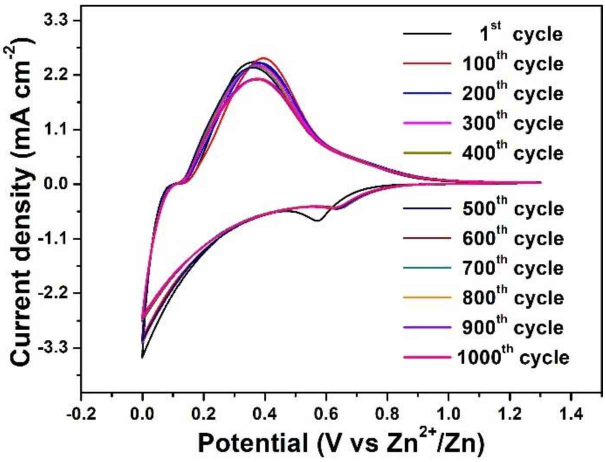

Fig. S14 Voltammograms of the electrochromic device at 20 mV s^{-1} in the 0-1.3 V window at 1st, 100th, up to 1000th cycles

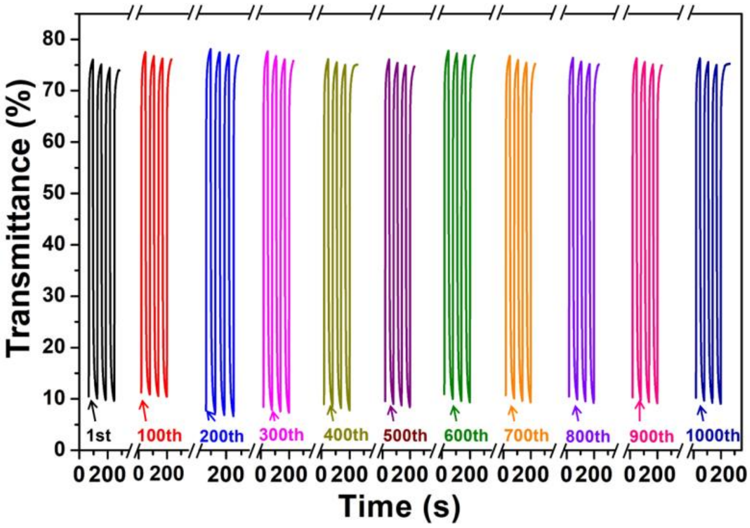

Fig. S15 Real-time transmission spectrum of the device during the cycling process

Table S4 Coloration and bleaching time of the device for 1st, 100th, 200th, up to 1000th cycle

Cycles	τ_c (s)	τ_b (s)
1st	9	2.7
100th	8.6	4.7
200th	9.7	4.7
300th	9.2	4
400th	10.8	4
500th	10.4	4.6
600th	11.4	5.1
700th	10.8	4.1
800th	9.3	3.6
900th	9.7	3.2
1000th	10.3	3.9

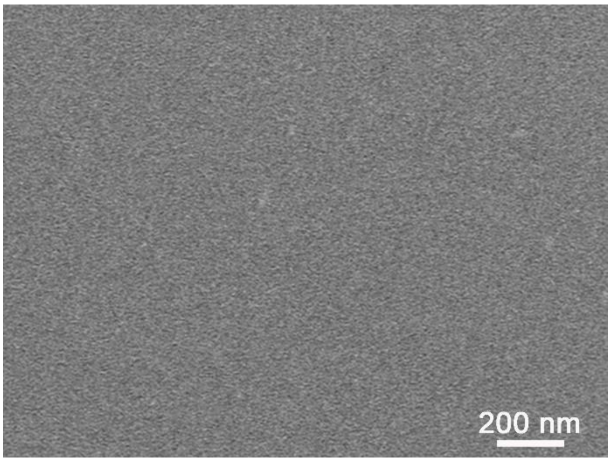

Fig. S16 SEM image of W4 NC film disassembled from device after 1000 cycles

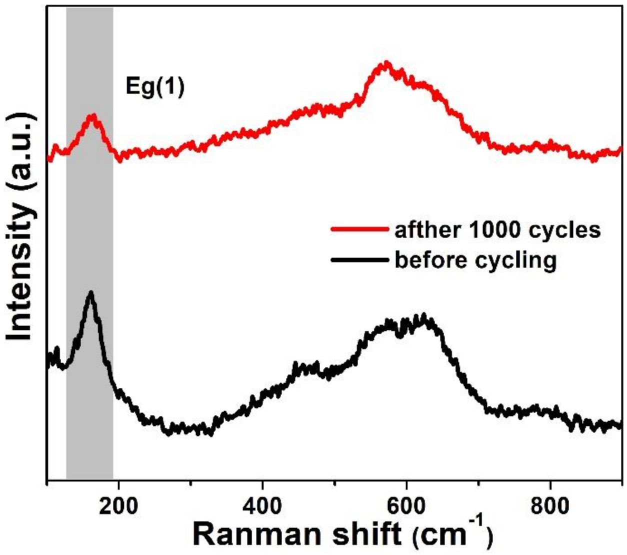

Fig. S17 Raman spectra of the W4 electrochromic device before and after 1000 cycles

Table S5 Performance comparison of Zn^{2+}-based electrochromic device

year	material	electrolyte	response time (s)	optical modulation (%)	cycle stability (transmittance reduction)	Refs.
2019	Ti-substituted tungsten molybdenum oxide nanowire bundles	1 M $ZnSO_4$	——	62 (at 633 nm)	31% decayed after 100 cycles	[S9]
2019	WO_3 nanoparticles	1 M $ZnSO_4$-$AlCl_3$	τ_b/τ_c:5.7/10.3 (at 633 nm)	77 (at 633 nm)	43% decayed after 200 cycles	[S10]
2019	V_3O_7 nanoparticles	1 M $ZnSO_4$	τ_b/τ_c:10.4/28.6 (at 633 nm)	21 (at 633 nm)	——	[S19]
2020	WO_{3-x} nanocrystals	1 M $ZnSO_4$	τ_b/τ_c:4.5/3.7 (at 633 nm)	76 (at 633 nm)	——	[S20]
2020	sodium vanadium oxide nanorods	1 M $ZnSO_4$	τ_b/τ_c:23.2/34.8 (at 633 nm)	21 (at 633 nm)	38% decayed after 1000 cycles	[S11]
2020	WO_3 nanorods	1 M $ZnSO_4$	τ_b/τ_c:7.4/7.2 (at 550 nm) τ_b/τ_c:4.7/12 (at 633 nm)	66 (at 550 nm) 75 (at 633 nm)	33% decayed after 500 cycles	[S5]
2021	W-doped TiO_2 nanocrystals	1 M $ZnSO_4$	τ_b/τ_c:2.7/9 (at 550 nm)	66 (at 550 nm)	8.7% decayed after 1000 cycles	This work

Supplementary References

[1] S. Cao, S. Zhang, T. Zhang, J.Y. Lee, Fluoride-assisted synthesis of plasmonic colloidal Ta-doped TiO_2 nanocrystals for near-infrared and visible-light selective electrochromic modulation. Chem. Mater. **30,** 4838-4846 (2018). https://doi.org/10.1021/acs.chemmater.8b02196

[2] S. Zhang, S. Cao, T. Zhang, J.Y. Lee, Plasmonic oxygen-deficient TiO_{2-x} nanocrystals for dual-band electrochromic smart windows with efficient energy recycling. Adv. Mater. **32,** e2004686 (2020). https://doi.org/10.1002/adma.202004686

[3] T.R. Gordon, M. Cargnello, T. Paik, F. Mangolini, R.T. Weber et al., Nonaqueous synthesis of TiO_2 nanocrystals using TiF_4 to engineer morphology, oxygen vacancy concentration, and photocatalytic activity. J. Am. Chem. Soc. **134,** 6751-6761 (2012). https://doi.org/10.1021/ja300823a

[4] X. Wu, Z. Chen, G.Q.M. Lu, L. Wang, Nanosized anatase TiO_2 single crystals with tunable exposed (001) facets for enhanced energy conversion efficiency of

dye-sensitized solar cells. Adv. Funct. Mater. **21,** 4167-4172 (2011). https://doi.org/10.1002/adfm.201100828

[5] Q. Huang, S. Cao, Y. Liu, Y. Liang, J. Guo et al., Boosting the Zn^{2+}-based electrochromic properties of tungsten oxide through morphology control. Sol. Energy Mater. Sol. Cells **220,** 110853 (2021). https://doi.org/10.1016/j.solmat.2020.110853

[6] R. Marder, R. Chaim, C. Estournès, Grain growth stagnation in fully dense nanocrystalline Y_2O_3 by spark plasma sintering. Mater. Sci. Eng. A **527,** 1577-1585 (2010). https://doi.org/10.1016/j.msea.2009.11.009

[7] J. Wallot, P. Reynders, A.A. Herzing, C.J. Kiely, M.P. Harmer et al., Sintering of thin film nanocrystalline titania–tin oxide composites. J. Eur. Ceram. Soc. **28,** 2225-2232 (2008). https://doi.org/10.1016/j.jeurceramsoc.2008.02.019

[8] Z.Z. Fang, X. Wang, T. Ryu, K.S. Hwang, H.Y. Sohn, Synthesis, sintering, and mechanical properties of nanocrystalline cemented tungsten carbide – A review. Int. J. Refract. Met. Hard Mater. **27,** 288-299 (2009). https://doi.org/10.1016/j.ijrmhm.2008.07.011

[9] H. Li, L. McRae, C.J. Firby, A.Y. Elezzabi, Rechargeable aqueous electrochromic batteries utilizing Ti-substituted tungsten molybdenum oxide based Zn^{2+} ion intercalation cathodes. Adv. Mater. **31,** e1807065 (2019). https://doi.org/10.1002/adma.201807065

[10] H. Li, C.J. Firby, A.Y. Elezzabi, Rechargeable aqueous hybrid Zn^{2+}/Al^{3+} electrochromic batteries. Joule **3**, 2268-2278 (2019). https://doi.org/10.1016/j.joule.2019.06.021

[11] W. Zhang, H. Li, W.W. Yu, A.Y. Elezzabi, Transparent inorganic multicolour displays enabled by zinc-based electrochromic devices. Light Sci. Appl. **9,** 121 (2020). https://doi.org/10.1038/s41377-020-00366-9

[12] M. Barawi, L. De Trizio, R. Giannuzzi, G. Veramonti, L. Manna, M. Manca, Dual band electrochromic devices based on Nb-doped TiO_2 nanocrystalline electrodes. ACS Nano **11,** 3576-3584 (2017). https://doi.org/10.1021/acsnano.6b06664

[13] S. Cao, S. Zhang, T. Zhang, Q. Yao, J.Y. Lee, A visible light-near-infrared dual-band smart window with internal energy storage. Joule **3,** 1152-1162 (2019). https://doi.org/10.1016/j.joule.2018.12.010

[14] K.R. Reyes-Gil, Z.D. Stephens, V. Stavila, D.B. Robinson, Composite WO_3/TiO_2 nanostructures for high electrochromic activity. ACS Appl. Mater. Interfaces **7,** 2202-2213 (2015). https://doi.org/10.1021/am5050696

[15] T. Dhandayuthapani, R. Sivakumar, D. Zheng, H. Xu, R. Ilangovan et al., WO_3/TiO_2 hierarchical nanostructures for electrochromic applications. Mater.

Sci. Semicond. Proc. **123**(2021) 105515. https://doi.org/10.1016/j.mssp.2020.105515

[16] X. Zhang, F. Liu, Q.-L. Huang, G. Zhou, Z.-S. Wang, Dye-sensitized W-doped TiO_2 solar cells with a tunable conduction band and suppressed charge recombination. J. Phys. Chem. C **115,** 12665-12671 (2011). https://doi.org/10.1021/jp201853c

[17] J. Guo, X. Guo, H. Sun, Y. Xie, X. Diao et al., Unprecedented electrochromic stability of a-WO_{3-x} thin films achieved by using a hybrid-cationic electrolyte. ACS Appl. Mater. Interfaces **13,** 11067-11077 (2021). https://doi.org/10.1021/acsami.0c22921

[18] D.-M. Chen, G. Xu, L. Miao, L.-H. Chen, S. Nakao et al., W-doped anatase TiO_2 transparent conductive oxide films: theory and experiment. J. Appl. Phys. **107,** 063707 (2010). https://doi.org/10.1063/1.3326940

[19] W. Zhang, H. Li, M. Al-Hussein, A.Y. Elezzabi, Electrochromic battery displays with energy retrieval functions using solution-processable colloidal vanadium oxide nanoparticles. Adv. Opt. Mater. **8** 1901224 (2019). https://doi.org/10.1002/adom.201901224

[20] L. Zhang, D. Chao, P. Yang, L. Weber, J. Li et al., Flexible pseudocapacitive electrochromics via inkjet printing of additive-free tungsten oxide nanocrystal ink, Adv. Energy Mater. **10,** 2000142 (2020). https://doi.org/10.1002/aenm.202000142
